# Pharmacokinetic Study and Metabolite Identification of CAM106 in Rats by Validated UHPLC-MS/MS

**DOI:** 10.3390/ph16050728

**Published:** 2023-05-11

**Authors:** Ruqi Xi, Rahima Abdulla, Jiangyu Zhao, Haji Akber Aisa, Yongqiang Liu

**Affiliations:** 1 State Key Laboratory Basis of Xinjiang Indigenous Medicinal Plants Resource Utilization, Xinjiang Technical Institute of Physics and Chemistry, Chinese Academy of Sciences, Urumqi 830011, China; xiruqi20@mails.ucas.ac.cn (R.X.); haji@ms.xjb.ac.cn (H.A.A.); 2 University of Chinese Academy of Sciences, No. 19(A) Yuquan Road, Shijingshan District, Beijing 100049, China

**Keywords:** CAM106, pharmacokinetic, metabolites, LC–MS/MS, rats

## Abstract

Given the limitations of existing antiviral drugs and vaccines, there is still an urgent need for new anti-influenza drugs. CAM106, a rupestonic acid derivative, was studied for its potent antiviral activity and showed a favorable inhibitory effect on influenza virus replication. However, many gaps exist in preclinical studies of CAM106. This study focused on the pharmacokinetic profile and metabolites of CAM106 in vivo. An efficient and fast bioanalytical method was successfully developed and validated for the quantitation of CAM106 in rat plasma. A mobile phase aqueous solution (A, containing 0.1% formic acid) and acetonitrile (B) worked within 0–3.5 min, with 60% B. The mass spectrum scanning mode was the parallel reaction monitoring (PRM) with a resolution of 17,500. The linear range of the method was 2.13–1063.83 ng/mL. The validated method was applied to a pharmacokinetic study in rats. The matrix effects ranged from 93.99% to 100.08% and the recovery ranged from 86.72% to 92.87%. The intra- and inter-day precisions were less than 10.24% and the relative error (RE) ranged from −8.92% to 7.1%. The oral bioavailability of CAM106 was 1.6%. Thereafter, its metabolites in rats were characterized using high-resolution mass spectrometry. The isomers M7-A, M7-B, M7-C, and M7-D were well separated. As a result, a total of 11 metabolites were identified in the feces, urine, and plasma of rats. The main metabolic pathways of CAM106 were oxidation, reduction, desaturation, and methylation. The assay was reliable and provided useful information for further clinical studies of CAM106.

## 1. Introduction

Viruses, tiny in size and simple in structure, are non-cellular organisms [1,2,3]. Virus replication, transcription, and translation must take place in a host cell [4,5,6]. A virus can produce viral particles and use nutrients from the host cell to reproduce by replicating its own nucleic acids and proteins autonomously [7,8]. Infectious diseases caused by viruses are highly contagious, with high morbidity and mortality rates, seriously endangering human life and health [9,10,11]. Influenza viruses are single-stranded, negative-strand RNA viruses that cause influenza in humans and animals [12,13]. Taxonomically, influenza viruses belong to the family Orthomyxoviridae [14]. They cause acute upper respiratory infections and spread rapidly by air, resulting in periodic pandemics throughout the world [15,16,17]. Throughout human history, there have been several public health crises caused by influenza viruses, such as the Spanish influenza (H1N1) in 1918 [18], Asian influenza (H2N2) in 1957 [19], Hong Kong influenza (H3N2) in 1968 [20], and influenza A in 2009 [21]. Each pandemic outbreak caused enormous damage to the economies of the affected countries and to human health [22].

**Figure 1:**
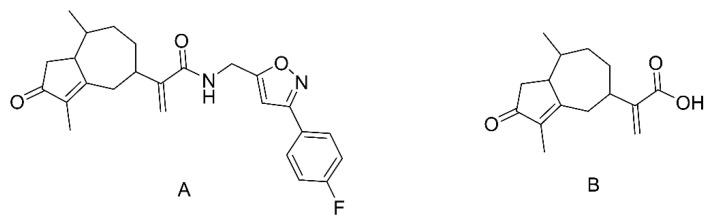
Chemical structures of CAM106 (**A**) and rupestonic acid (**B**).

Among a series of synthesized rupestonic acids, CAM106 (Figure 1A) exhibits excellent antiviral activity. The half-maximal inhibitory concentrations (IC_50_) of CAM106 for the H3N2, H1N1, and B viruses are 1.09, 0.97, and 3.25 μmol/L, respectively [23]. In addition, CAM106 suppresses influenza viruses’ replication by activating the heme oxygenase−1−mediated interferon response [24]. This shows that CAM106 is an excellent lead compound against influenza viruses. 

*Artemsia rupestris* L. (*A. rupestris)* is a plant of the genus *Artemisia* of the Compositae family [25]. The main chemical components of *A. rupestris* are sesquiterpenes, flavonoids, alkaloids, dimers, and volatiles, among which, sesquiterpenes have better antiviral activity than that of other components [26,27]. One of the *A. rupestris* components contains 8.79 mg/kg rupestonic acid and shows great anti-influenza-virus activity. The IC_50_ of rupestonic acid for virus B is 115.7 μmol/L [28,29]. To further improve the antiviral activity of rupestonic acid, our laboratory modified the structure of rupestonic acid, and compound CAM106 was synthesized. The IC_50_ of CAM106 against influenza virus B was about 35-fold higher than that of rupestonic acid. However, preclinical studies of CAM106 are still ongoing and its pharmacokinetic profiles and metabolites remain unstudied.

The purposes of this study were to determine the bioavailability of CAM106 by both oral and intravenous administration and to characterize the metabolites of CAM106 in the feces, urine, and plasma of rats. Rupestonic acid was used as an internal standard (IS, Figure 1B). A rapid, selective, effective, and reliable assay for the measurement of CAM106 in rat plasma was developed and validated. High-resolution mass spectrometry was used for the identification of CAM106 metabolites. Finally, the pharmacokinetic parameters of CAM106 and its metabolic pathway in rats were determined.

## 2. Results and Discussion

This study developed a highly selective and efficient bioassay for the accurate quantitative analysis of CAM106 in rats. The 11 metabolites of CAM106 in rats were characterized by high-resolution mass spectrometry.

To optimize the elution conditions, different organic solvents, including acetonitrile and methanol, were investigated. We first intended to use methanol as the organic solvent. However, we found that methanol caused obvious column pressure and had a weak elution ability. Hence, acetonitrile was used to avoid overpressure of the LC system. In addition, 0.1% formic acid was employed as the aqueous phase modifier to promote ionization. Finally, the mobile phase aqueous solution (A, containing 0.1% formic acid) and acetonitrile (B) worked within 0–3.5 min, with 60% B. To optimize the MS/MS conditions, both positive and negative ESI modes were tested for mass spectra, and the positive mode was selected because CAM106 and IS showed strong [M+H]^+^ responses. The optimized precursor-to-product ion transition monitored for CAM106 was an *m*/*z* of 423.21/177.13, while IS was monitored at an *m*/*z* of 249.15/107.09. To optimize the protein precipitation method, acetonitrile had the advantage over liquid–liquid extraction and solid-phase extraction by being comparatively simple and inexpensive.

### 2.1. Method Validation

#### 2.1.1. Specificity

**Figure 2:**
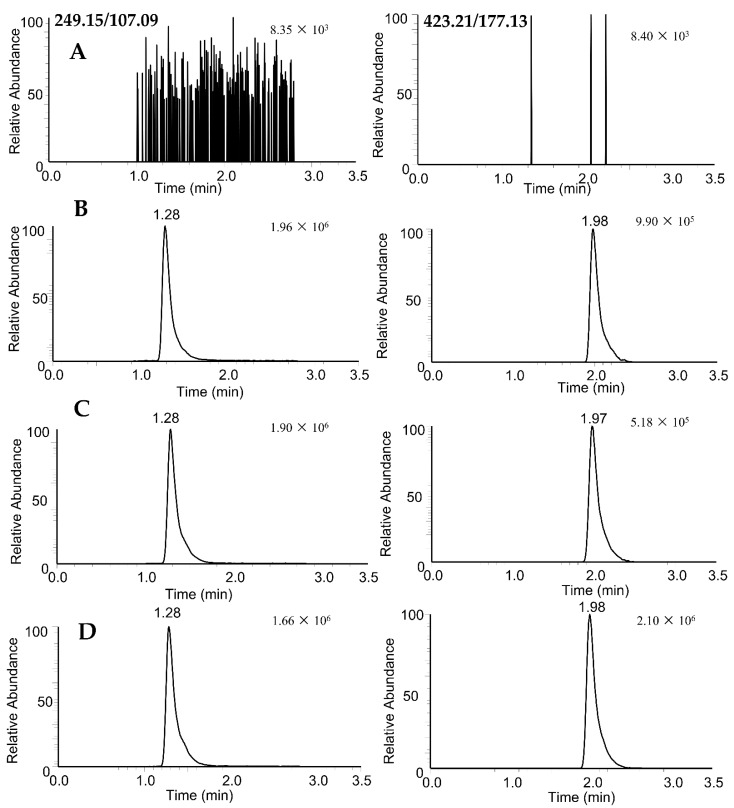
PRM extracted ion chromatograms of rupestonic acid (249.15/107.09) and CAM106 (423.21/177.13) in rat plasma: blank rat plasma (**A**); blank rat plasma spiked with 100 ng/mL of CAM106 and 319.15 ng/mL of internal standard (**B**); 1.5 h after oral administration of CAM106 (**C**); and 0.5 h after intravenous administration of CAM106 (**D**).

No interfering peaks at the retention times of CAM106 and IS were observed in the different blank rat plasma samples. All of the plasma batches chosen showed suitable selectivity and minimal background interference. The results are shown in Figure 2.

#### 2.1.2. Calibration Curves

The calibration curves were established by plotting the peak area ratios of CAM106 to IS concentrations. The standard curve showed good linearity at different concentrations over the range of 2.13–1063.83 ng/mL, and the formula was:

Y = −0.00131731 + 0.0017645 × X (r^2^ = 0.997)

#### 2.1.3. Accuracy and Precision

The plasma samples at four concentrations were tested for intra- and inter-day variability for evaluating the precision and accuracy of the method. As shown in Table 1, the intra- and inter-day precisions (RSD) of CAM106 were less than 10.24 %, and the accuracy (RE) ranged from −8.92% to 7.11%. The results showed that the developed method was accurate and reliable in the analysis of plasma samples.

#### 2.1.4. Dilution Integrity

Dilution integrity was evaluated by a 4-fold dilution of an analyte stock solution (3000 ng/mL). The result of the dilution was 714.90 ± 18.59 ng/mL. This result shows that the accuracy of dilution is within the criteria.

#### 2.1.5. Stability

Three quality control (QC) concentrations of CAM106 were used for stability testing under five conditions: in the autosampler for 0, 4, 8, and 12 h; long-term stability at −80 °C for 1 and 7 days; stock solution for 15 days at −20 °C; at room temperature for 12 h; and three freeze–thaw cycles. The stability investigation results of CAM106 in plasma are shown in Table 2.

#### 2.1.6. Recovery and Matrix Effect

The extraction recoveries were consistent and concentration-independent at the three QC concentration levels. There was no significant matrix enhancement or suppression effect on the analyte. The results are shown in Table 3. The recovery and matrix effects of CAM106 from plasma were 86.72–92.87% and 97.89–100.08%, respectively.

### 2.2. Pharmacokinetic (PK) Analysis and PK Parameter

**Figure 3:**
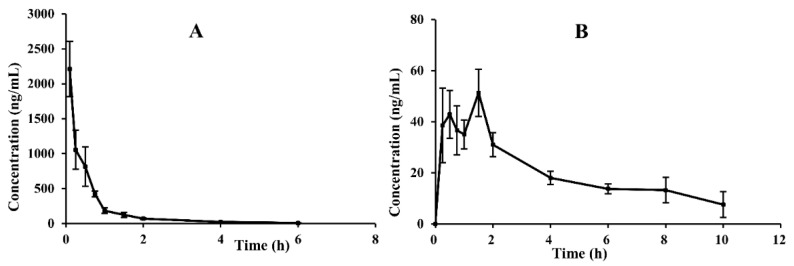
Mean plasma concentration–time curves of CAM106 in rats after oral (180.0 mg/kg; n = 6, mean ± SEM) (**A**) and intravenous administration (18.0 mg/kg; n = 6, mean ± SEM) (**B**).

No adverse reactions or acute toxicity were observed after oral (180 mg/kg) or intravenous (18 mg/kg) administration of CAM106 in rats. By using the UHPLC–MS/MS method, we successfully performed accurate quantification of CAM106 in rat plasma, and a non-compartment analysis was used to characterize the pharmacokinetic profiles of CAM106. Figure 3 shows the profiles of plasma drug concentrations over time after oral and intravenous administration of CAM106. Table 4 shows the pharmacokinetic parameters of CAM106 in rats. After oral administration, the C_max_ of CAM106 was found to be 61.03 ± 30.81 ng/mL at a T_max_ of 1.21 ± 0.51 h. After intravenous administration, T_1/2_ and V_d_ were 1.34 ± 0.64 h and 16624.89 ± 8171.11 mL/kg, respectively. Additionally, the oral bioavailability of CAM106 was 1.60%. This indicated that the exposure of CAM106 in vivo after oral administration was extremely low. The mean retention times after oral and intravenous administration were 3.42 ± 0.73 h and 0.63 ± 0.17 h, respectively. This showed that CAM106 has a long absorption time.

#### 2.2.1. Fragmentation of CAM106

**Figure 4:**
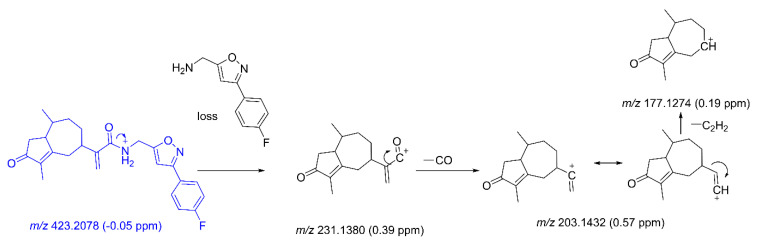
The characteristic fragments of CAM106.

The metabolites were derivatives of CAM106. Some of the metabolite fragments overlapped with fragments of CAM106. Therefore, it is necessary to identify the characteristic fragments of CAM106. The retention time of CAM106 was 27.15 min, and its protonated molecule with *m*/*z* 423.2078 (−0.05 ppm) was detected. The major fragments had *m*/*z* values of 231.1380, 203.1432, and 177.1274. The fragment of the *m*/*z* 231.1380 ion was generated by the amide bond rupture of CAM106. It was further broken into pieces with *m*/*z* values of 203.1432 and 177.1274 by the loss of CO and C_2_H_2_, respectively. Figure 4 shows the cleavage mechanism for CAM106.

#### 2.2.2. Metabolites of CAM106

**Figure 5:**
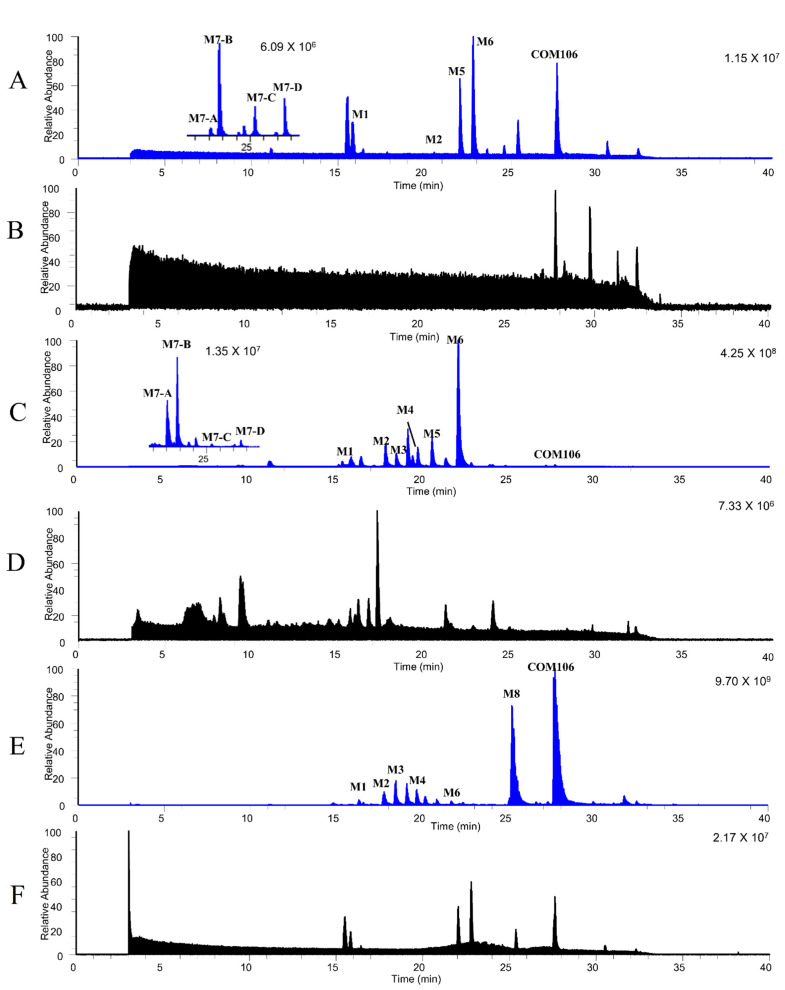
Parallel reaction monitoring (PRM) chromatograms of CAM106 and its metabolites in rats. The PRM chromatogram of CAM106 and its metabolites in plasma samples (**A**), blank plasma samples (**B**), urine samples (**C**) blank urine samples (**D**), feces samples (**E**), and blank feces samples (**F**).

**Figure 6:**
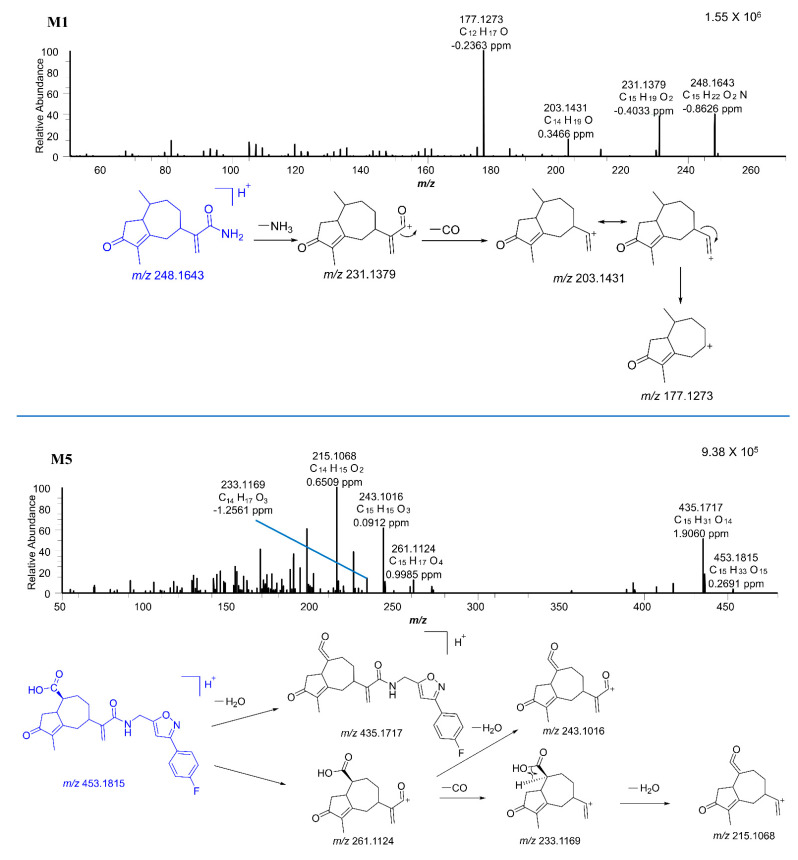
The MS/MS spectra of M1 and M5.

**Figure 7:**
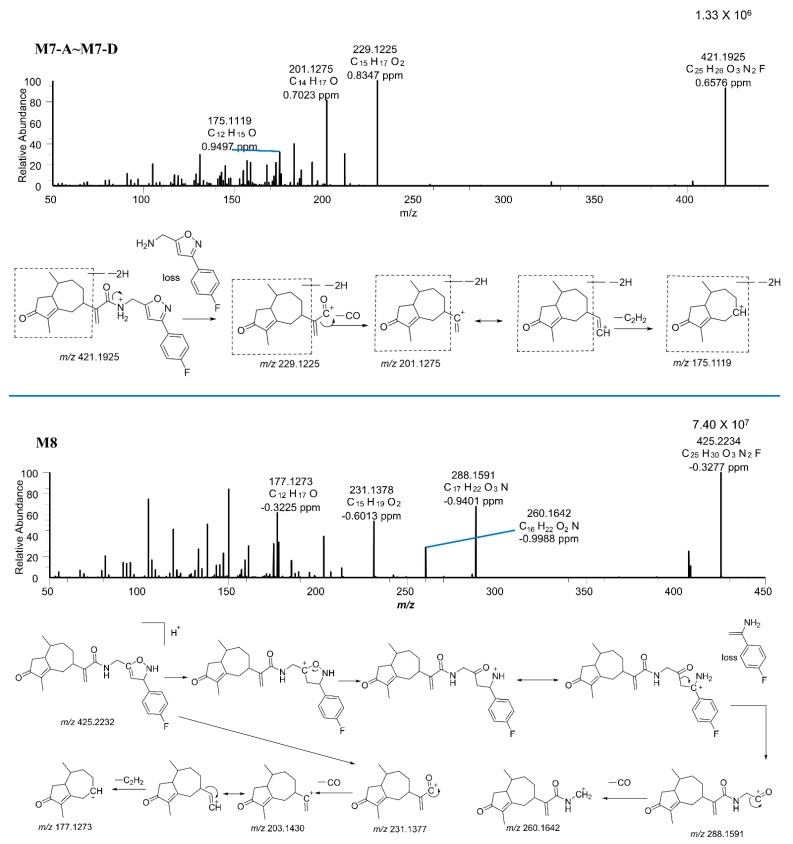
The MS/MS spectra of M7−A~M7−D, and M8.

CAM106 metabolites were identified in the rat’s urine, feces, and plasma. After comparison between blank and administered samples, a total of 11 metabolites were identified. Figure 5 shows the combined parallel reaction monitoring (PRM) chromatogram of CAM106 and its metabolites. Accurate mass measurements and key fragmental ions of metabolites and CAM106 are shown in Table 5. Figure 6 shows the fragmentation behavior of M1 and M5. Figure 7 shows the fragmentation behavior of M7−A~M7−D and M8.

M5 was observed at 20.98 min with a protonated molecular weight of *m*/*z* 453.1815 (0.26 ppm). The major fragment ions of M5 had *m*/*z* values of 435.1717, 261.1124, 243.1016, 233.1169, and 215.1068. The *m*/*z* 435.1717 fragment ion was generated from M5 by the loss of H_2_O. The *m*/*z* 261.1124 fragment ion was produced by the breakage of the M5 amide bond. It was further broken into pieces with *m*/*z* values of 243.1016 and 233.1169 due to the loss of H_2_O and CO, respectively. Additionally, the fragment ion with an *m*/*z* of 215.1068 was generated from the *m*/*z* 233.1169 ion by loss of H_2_O.

M7–A, M7–B, M7-C, and M7–D were observed at 22.64, 24.57, 25.38, and 27.56 min, respectively, with protonated molecular weights of *m*/*z* 421.1925 (0.84 ppm), 421.1927 (−1.31 ppm), 421.1930 (1.96 ppm), and 421.2027 (1.16 ppm). The spectra indicated that CAM106 was desaturated at four different positions. Since the characteristic fragments of M7–A, M7–B, M7–C, and M7-D were the same, M7–A was used as an example to illustrate the cleavage pattern. The major fragment ions of M7–A had *m*/*z* values of 229.1225, 201.1275, and 175.1119. The *m*/*z* 229.1225 fragmentation ion was generated from amide bond breaking. It was further fragmented into two pieces with *m*/*z* values of 201.1275 and 175.1119 due to the loss of CO and C_2_H_2_, respectively. 

M8 showed a precursor ion at *m*/*z* 425.2234 (−0.33 ppm) with a retention time of 25.13 min. The MS/MS spectra of M8 were quite similar to those of CAM106, such as peaks with *m*/*z* values of 231.1377, 203.1430, and 177.1273. However, there were two relatively abundant fragment ions with *m*/*z* values of 288.1591 and 260.1642 that were not present in the CAM106 spectra. The *m*/*z* 288.1591 fragment ion was produced by the cleavage of the isoxazole ring. It was a further neutral loss of CO that produced the *m*/*z* 260.1642 fragment ion. This was the first study to investigate the metabolism of CAM106 in rats. 

**Figure 8:**
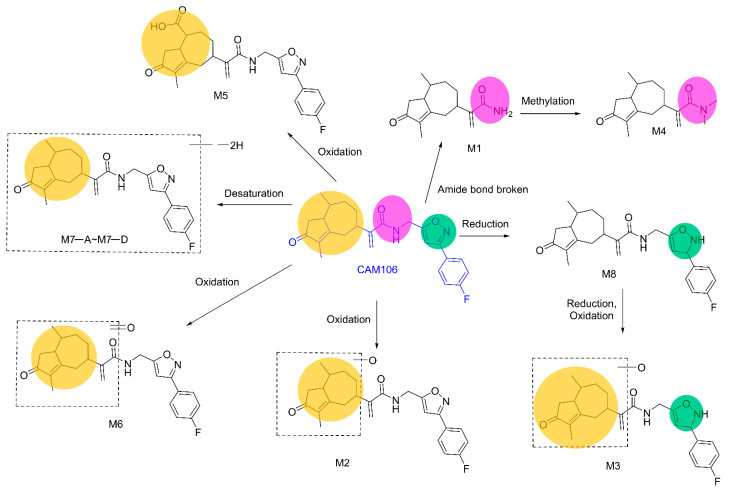
The proposed metabolic pathways of CAM106.

The proposed metabolic pathways of CAM106 are shown in Figure 8. A total of 11 metabolites were identified in feces, urine, and plasma. The major metabolic pathways included reduction, oxidation, desaturation, and methylation. CAM106 mainly underwent phase I metabolism. For phase II metabolism, only methylation was detected in this study.

#### 2.2.3. Discussion

Because of the superior antiviral activity of CAM106, it is still an interesting lead candidate for treatments against influenza viruses. Although the oral bioavailability of CAM106 in rats is only 1.6%, it can be improved by different formulations. Some components in traditional Chinese medicine such as flavonoids, etc., present low oral bioavailability. However, different formulations could improve oral bioavailability [30]. Moreover, the compound CAM106 is poorly soluble in water, so the formulation particle size can be changed to improve bioavailability. CAM106’s specific surface area can be increased by nano-formulation which is used to facilitate the dissolution of drugs. CAM106 can also be made into a hydrochloride form to improve its bioavailability [31].

## 3. Materials and Methods

### 3.1. Reagents and Chemicals

CAM106 (99.4%) was synthesized by the Xinjiang Technical Institute of Physics and Chemistry, Chinese Academy of Sciences (Urumqi, China) [27]. Rupestonic acid (99.2%, Figure 1, IS) was isolated by Xinjiang Technical Institute of Physics and Chemistry, Chinese Academy of Sciences (Urumqi, China). UHPLC–MS/MS-grade acetonitrile, methanol, and formic acid were obtained from Thermo Fisher Scientific (Bremen, Germany). Blank rat plasma was purchased from Wuhan Purity Biotechnology Company (Wuhan, China). Ethanol was purchased from Tianjin Xin Platinum Chemical Company (Tianjin, China). Distilled water was purchased from Watsons (Guangzhou, China). Kolliphor EL (PEG-35, CAS, 61791-12-6) was obtained from Sigma–Aldrich (St. Louis, MO, USA).

### 3.2. Animals and Experiments

All twelve male Sprague Dawley (SD) rats (weight 200 ± 20 g) were purchased from Xinjiang Medical University (Xinjiang, China). Due to CAM106’s weak water solubility, it was dissolved in a mixture of 2% ethanol, 8% Kolliphor, and 90% distilled water. For the pharmacokinetic study, twelve rats were randomized into two groups (n = 6). The rats were fasted overnight before the experiment but had free access to water. CAM106 showed no toxic response when given to Kun Ming mice at 25 mg/kg [28]. Rats were given 180 mg/kg (for oral administration) and 18 mg/kg (for intravenous administration) of CAM106 for 3 days and no signs of toxicity were observed. For the oral administration group, CAM106 was intragastrically administered to the rats at a dose of 180 mg/kg. Approximately 0.2 mL of blood was drawn from the ophthalmic veins at 0, 0.25, 0.5, 0.75, 1, 1.5, 2, 4, and 10 h. For intravenous administration, CAM106 was injected into the tail at a dose of 18 mg/kg. About 0.2 mL of blood (from the ophthalmic veins) was drawn at 0, 0.25, 0.5, 0.75, 1, 1.5, 2, 4, and 6 h. After that, 1.5 mL polyethylene tubes containing EDTA were used to collect the plasma. The blood samples were centrifuged at 19,800× *g* for 7 min after which the supernatant was collected and stored at −80 °C for further analysis.

### 3.3. Calibration Standard 

Stock standard solutions at 1.0 mg/mL CAM106 and internal standard were prepared with methanol. The calibration solution was prepared by adding the appropriate amount of working solution to the blank rat plasma. The final concentration range was 2.13–1063.83 ng/mL for CAM106. 

### 3.4. Preparation of Quality Control Samples

A 10 µL volume of CAM106 was added to 100 µL of the plasma samples and vortexed for 3 s (3000 rpm). Then, the mixtures were vortexed for three seconds while receiving 10 µL of internal standard (3000 rpm). A volume of 350 µL acetonitrile was added to 100 µL of each plasma sample. The mixtures were vortexed for 1 min and centrifuged for 7 min at 19,800 g. The final concentrations of the QC samples produced were 10.64, 106.38, and 638.30 ng/mL. The final concentration was 319.15 ng/mL for IS. 

### 3.5. Pharmacokinetic Sample Preparation

A 10 µL volume of methanol was added to 100 µL of each plasma sample and vortexed for 3 s (3000 rpm). Then, the mixtures were vortexed for three seconds while receiving 10 µL of internal standard (3000 rpm). A volume of 350 µL acetonitrile was added to 100 µL of each plasma sample. The mixtures were vortexed for 1 min and centrifuged for 7 min at 19800 g. A 100 µL aliquot of the supernatant was stored and 5 µL was subjected to UHPLC–MS/MS analysis.

### 3.6. Instruments and UHPLC-MS/MS Conditions

The Dionex UltiMate 3000 RSLCnano System and Thermo Scientific Q Exactive Plus Orbitrap were used for the UHPLC-MS/MS bioanalytical technique. Thermo Xcalibur 4.2.28.14 Quan Browser was used to process the data while Xcalibur controlled the system (Thermo Fisher Scientific, Waltham, MA, USA).

For the pharmacokinetic study, the chromatographic separation was carried out on an Acquity UHPLC BEH C_18_ column (2.1 × 75 mm, 1.7 µm) with a filter. The column oven was set at 35 °C. The mobile phase aqueous solution (A, containing 0.1% formic acid) and acetonitrile (B) worked within 0–3.5 min with 60% B. The injection volume was set at 5 µL, with a flow rate of 0.2 mL/min. By using the parallel reaction monitoring (PRM) mode, the CAM106 and internal standard were found. The following adjustments were made to the electrospray ionization (ESI) source parameters: high collision dissociation cell (HCD) energy, 27 eV (for CAM106) and 42 eV (for the IS); capillary temperature, 320 °C; S-lens RF level, 55 V; automatic gain control (AGC) target, 1e^6^; maximum IT, 100 ms; spray voltage, 3.2 kV.

For the metabolic study, the instrument system used and the instrument parameters were the same as above. The HCD energy was normalized collision energy (NCE): 20, 40, and 60 eV. With a resolution of 17500, the complete MS/dd MS2 mode was used to find the metabolites. The mobile phase aqueous solution (A), which contains 0.1% formic acid, and acetonitrile (B) worked as follows: 0–3 min (5% B), 3–23 min (40% B), 23–30 min (80% B), 30–31 min (100% B), 31–40 min (100% B).

### 3.7. Method Validation

The method was validated according to the United States Food and Drug Administration Bioanalytical Method Validation Guidance for Industry [32].

#### 3.7.1. Specificity

The selectivity of the method was evaluated using rat blank plasma samples from six different rats to assess interference from any interfering endogenous components in the plasma. Blank samples did not show peaks at the CAM106 and IS retention times. Additionally, the methodologically validated, orally administered, and intravenously injected samples showed peaks at the CAM106 and IS retention times. This indicates that the specificity of the method is good. 

#### 3.7.2. Calibration Curves

To construct the calibration curves, the ratio of the peak area (CAM106/IS) was plotted against the nominal calibration standard concentrations using the weighted least squares linear regression method (1/x^2^). The standard calibration curve was accepted if the back-calculated concentrations for at least 75% of the calibration curve were within ±15% of their theoretical concentrations, except for the lower limit of quantification (±20%). The data were processed using Thermo Xcalibur 4.2.28.14 Quan Browser.

#### 3.7.3. Accuracy and Precision

To evaluate the intra- and inter-day precision and accuracy, three consecutive analytical batches including four concentrations of QC samples (2.13, 10.64, 106.38, and 638.30 ng/mL) with six replicates for CAM106 were performed. The accuracy and precision were calculated using the relative error (RE) and relative standard deviation (RSD).

#### 3.7.4. Dilution Integrity

The integrity of the dilutions was tested to ensure that the dilution of the samples had no effect on the accuracy of the measurements. The CAM106 stock solution was added to the blank plasma to prepare a 3000 ng/mL working stock. Six replicates of this concentration were prepared. Blank rat plasma was added to dilute it 4-fold and its accuracy was assessed. 

#### 3.7.5. Stability

The stability tests were conducted with rat plasma containing CAM106 through the analysis of 3 replicates at 10.64, 106.38, and 638.30 ng/mL. This typically comprises short-term stability (room temperature), long-term stability (−80 °C), post-preparation stability (autosampler, 10 °C), and freeze–thaw cycle stability (room temper and −80 °C). Acceptable results were obtained for the stability tests, as the relative error was less than 15% of the normal concentration, which demonstrated the stability of the treated samples.

#### 3.7.6. Recovery and Matrix Effect

The extraction recoveries of CAM106 and IS were assessed by measuring the peak area ratios of analytes in pre-extraction spiked blank plasma to equal amounts of analytes. The extraction recovery of CAM106 and IS was evaluated by measuring six replicate plasma samples at 10.64, 106.38, 638.30, and 319.15 ng/mL. Matrix effects were assessed by determining the ratio of the peak area of analytes dissolved in the supernatant of treated blank plasma to the peak area of a pure working solution containing an equivalent amount of analytes. The matrix effects of CAM106 and IS were measured in six replicate plasma samples at 10.64, 106.38, 638.30, and 319.15 ng/mL. 

### 3.8. Metabolic Study 

For the plasma study, plasma collected at 0.5 h, 2 h, and 4 h after drug administration was added to 300 µL acetonitrile to remove the proteins. Afterwards, the samples were vortexed for 1 min (3000 rpm) and centrifuged at 19,800 g for 7 min. The supernatants were combined and blown dry under 40 °C nitrogen. A volume of 100 µL methanol was used to re-dissolve the sample and 5 µL was injected for analysis. The rats were fasted overnight with free access to water. Blank plasma was collected and stored at −80 °C. For blank sample preparation, three samples of 100 µL blank plasma were processed in the same way as the samples.

For the urine study, the urine was collected at 0–24 and 24–48 h after administration. In a 50 mL centrifuge tube, 5 mL of 0–24 h and 5 mL of 24–48 h urine were combine. A volume of 30 mL acetonitrile was added and the mixture was sonicated for 10 min. The samples were centrifuged for 10 min and the supernatant was blown dry under 40 °C nitrogen. A volume of 100 µL methanol was used to re-dissolve the sample and 5 µL was injected for analysis. The rats were placed in metabolic cages with a normal diet and water. Urine was collected at 0–24 h and 24–48 h and stored at −80 °C. For blank sample preparation, 10 mL of blank urine was processed in the same way as the samples.

For the feces study, the feces were collected at 0–24 and 24–48 h after administration. The feces were dried in the dark and ground into powder. In a 50 mL centrifuge tube, 4 mL of 0–24 h and 4 mL of 24–48 h feces were combined. A volume of 40 mL acetonitrile was added and the samples were sonicated for 30 min. The samples were centrifuged for 10 min and were blown dry under 40 °C nitrogen. A volume of 100 µL methanol was used to re-dissolve the sample and 5 µL was injected for analysis. The rats were placed in metabolic cages with a normal diet and water. Feces were collected at 0–24 h and 24–48 h and placed at room temperature to dry in the shade. The feces were ground into a powder and stored at −80 °C. For blank sample preparation, 4 mL of blank feces was processed in the same way as the samples.

## 4. Conclusions

The pharmacokinetics and metabolism of CAM106 were investigated in this study. In the pharmacokinetic study, CAM106 was slowly absorbed into the circulatory system and had a long elimination half-time. This indicated that the absorption rate could be improved by different modes of administration (sublingual, intramuscular). Additionally, the oral bioavailability was approximately 1.60%. CAM106 is still an interesting lead candidate due to its great anti-viral activity, even though it has low bioavailability. CAM106 can be made into nano formulations to increase the bioavailability by increasing the surface area to achieve increased contact area between the drug and the gastrointestinal mucosa. It can also be made into a hydrochloride form to improve its bioavailability. In the metabolism study, 11 metabolites were identified in feces, urine, and plasma. The major metabolic pathways of CAM106 were oxidation, reduction, desaturation, and methylation. Only methylation was detected in phase II, although sulfation and glucuronidation were the most common phase II metabolic pathways. Therefore, the focus should be on the phase II metabolites in future experiments with different species of animals.

## Figures and Tables

**Table 1:** Validation parameters: intra-day and inter-day accuracies and precisions of CAM106 in rat plasma (n = 6).

Analytes	Nominal Concentration (ng/mL)	Intra-Day Mean	RSD (%)	RE (%)	Inter-Day Mean	RSD (%)	RE (%)
CAM106	2.13	1.94	10.24	−8.92	2.07	6.63	−5.16
	10.64	10.60	8.95	−0.38	10.25	4.50	−3.67
	106.38	114.48	2.76	7.61	110.96	2.70	4.31
	638.30	645.41	1.52	7.11	630.82	4.44	−1.17

**Table 2:** Stability results of the validation parameters under different conditions.

Analytes	Nominal Concentration (ng/mL)	Accuracy								
CAM106		Autosampler stability				Long-term stability		Stock solution- 15 days	Room temperature	Thaw
		0 h	4 h	8 h	12 h	1 day	7 days			
	10.64	95.02	93.52	91.35	100.38	97.93	103.20	91.26	96.71	97.21
	106.38	106.05	101.96	101.46	98.88	104.02	94.03	108.39	101.12	106.13
	638.30	100.00	102.58	97.09	96.48	103.73	90.36	95.27	102.21	100.09

**Table 3:** The validation parameters: recovery and matrix effect of CAM106 and the internal standard in rat plasma.

Analytes	Nominal Concentration (ng/mL)	Recovery		Matrix Effect	
		Mean (%)	RSD (%)	Mean (%)	RSD (%)
CAM106	10.64	86.72	5.98	100.08	9.46
	106.38	92.61	13.75	98.23	2.97
	638.30	92.87	7.32	97.89	4.81
IS	319.15	96.55	6.71	93.99	5.53

**Table 4:** The main pharmacokinetic parameters of CAM106 in rats after intravenous and oral administration (n = 6, mean ± SD).

Parameters	Unit	PO * (180.0 mg/kg)	IV * (18.0 mg/kg)
AUC_0–t_ *	h·(ng/mL)	200.94 ± 87.45	1255.32 ± 544.72
AUC_0–inf_ *	h·(ng/mL)	294.19 ± 221.69	1287.93 ± 549.25
MRT_0–t_ *	h	3.42 ± 0.73	0.63 ± 0.17
MRT_0–inf_ *	h	6.69 ± 3.57	0.79 ± 0.23
T_max_ *	h	1.21 ± 0.51	—
T_1/2_ *	h	4.56 ± 2.31	1.34 ± 0.64
C_max_ *	ng/mL	61.03 ± 30.81	2313.88 ± 1004.00
CL*	mL/h/kg	799,138.27 ± 322,246.50	16,624.89 ± 8171.11
V_d_ *	mL/kg	4,605,807.05 ± 2,044,928.13	27,359.73 ± 10,321.12
F (%) *	1.60		

* AUC_0–t_ (AUC_0–inf_), area under the analyte concentration versus time curve from time 0 to t h (inf); MRT_0–t_ (MRT_0–inf_), mean residence time at time 0–t (inf); T_max_, the time of maximum concentration; T_1/2_, terminal half-life; C_max_, maximum concentration; CL, clearance; V_d_, apparent volume of distribution; F (%), absolute bioavailability; PO, peros; IV, intravenous.

**Table 5:** Characterization of CAM106 metabolites.

Metabolites	RT * (min)	Formula	Observed [M+H] ^+^	Calculated [M+H] ^+^	Error (ppm)	MS/MS	Transformation	Rat		
								P *	U *	F *
CAM106	27.15	C_25_H_27_FN_2_O_3_	423.2078	423.2078	0.02	405.1974, 231.1380, 203.1432,193.0773, 177.1274	Parent	√	√	√
M1	16.05	C_15_H_21_NO_2_	248.1643	248.1645	−0.79	231.1379, 203.1431, 177.1273	Broken amide bond	×	√	√
M2	19.07	C_25_H_27_FN_2_O_4_	439.2029	439.2028	0.06	421.1920, 245.1171, 217.1221, 178.0661	Oxidation	×	√	√
M3	19.33	C_25_H_29_FN_2_O_4_	441.2184	441.2184	−0.02	423.2081, 405.1989, 286.1436, 258.1488, 229.1233, 201.1275	Reduction, Oxidation	×	√	√
M4	19.56	C_17_H_25_NO_2_	276.1955	276.1958	−1.13	231.1380, 203.1431, 177.1274	Methylation	×	×	√
M5	20.98	C_25_H_26_O_5_N_2_F	453.1819	453.1820	−0.08	435.1717, 261.1124, 243.1016, 233.1169, 215.1068	Oxidation	√	√	×
M6	21.92	C_25_H_25_FN_2_O_4_	437.1859	437.1871	−2.85	419.1765, 245.1172, 203.1066, 199.1 117, 193.0773, 175.1120	Desaturation, Oxidation	√	√	√
M7−A	22.64	C_25_H_25_FN_2_O_3_	421.1925	421.1922	0.84	403.1831, 229.1223, 201.1274, 175.1118	Desaturation	√	√	×
M7−B	24.57	C_25_H_25_FN_2_O_3_	421.1927	421.1922	1.31	403.1827, 229.1226, 201.1276, 175.1127	Desaturation	√	√	×
M7−C	25.38	C_25_H_25_FN_2_O_3_	421.1930	421.1922	1.96	403.1833, 229.1227, 201.1227, 175.1119	Desaturation	√	√	×
M7−D	27.56	C_25_H_25_FN_2_O_3_	421.1927	421.1922	1.16	403.1837, 229.1227, 201.1227, 175.1120	Desaturation	√	√	×
M8	25.13	C_25_H_29_FN_2_O_3_	425.2219	425.2235	−3.69	407.2128, 231.1379 203.1431, 177.1273	Reduction	×	×	√

* P, plasma; U, urine; F, feces; RT, retention time.

## Data Availability

All data are contained in the article.
